# Does propofol ameliorate occurrence of postoperative cognitive dysfunction after general anaesthesia? A protocol of systematic review

**DOI:** 10.1186/s13643-021-01610-y

**Published:** 2021-03-18

**Authors:** Xi Zhao, Ze-qing Huang

**Affiliations:** Department of Anesthesiology, Cancer Hospital of China Medical University, Liaoning Cancer Hospital & Institute, No. 44 Xiao Heyan Road, Shenyang, 110042 China

**Keywords:** Propofol, Postoperative cognitive dysfunction, Anesthesia, Efficacy, Safety

## Abstract

**Background:**

Postoperative cognitive dysfunction (POCD) is a common condition after general anesthesia (GA). Previous studies have reported that propofol can ameliorate the occurrence of such disorder. However, its results are still inconsistent. Therefore, this systematic review will assess the efficacy and safety of propofol on POCD after GA.

**Methods:**

Literature sources will be sought from inception to the present in Cochrane Library, MEDLINE, EMBASE, PsycINFO, Web of Science, Scopus, Allied and Complementary Medicine Database, Chinese Biomedical Literature Database, and China National Knowledge Infrastructure for randomized controlled trials (RCTs) assessing the administration of propofol on POCD after GA. All searches will be carried out without limitations to language and publication status. Outcomes comprise of cognitive impairments changes, impairments in short-term memory, concentration, language comprehension, social integration, quality of life, and adverse events. Cochrane risk of bias tool will be utilized to assess study quality. We will evaluate the quality of evidence for each outcome using Grading of Recommendations Assessment, Development and Evaluation approach. A narrative synthesis or a meta-analysis will be undertaken as appropriate.

**Discussion:**

This study will systematically and comprehensively search literature and integrate evidence on the efficacy and safety of propofol on POCD after GA. Our findings will be of interest to clinicians and health-related policy makers.

**Systematic review registration:**

PROSPERO CRD42020164096

**Supplementary Information:**

The online version contains supplementary material available at 10.1186/s13643-021-01610-y.

## Background

Postoperative cognitive dysfunction (POCD) is one of the most common postoperative complications [1–3], which manifests as impairments in recent memory, concentration, language comprehension, and social integration [4, 5]. It is estimated that the incidence of POCD varies from 7 to 76% of surgical patients, especially in patients who are elderly [5, 6]. The 5-year mortality rate is about 70% due to POCD [7]. It often brings heavy health care burden for patient, their families, and society [8]. If it cannot be managed well, it may lead to substantial morbidity and mortality.

The occurrence of POCD is associated with surgical trauma and general anesthesia (GA) [9]. Of these, GA may play an essential role in detrimental effects on cognitive function via cholinergic system [10]. Previous studies have found that intravenous and inhaled anesthetics have neuroprotective effect [11, 12]. Propofol is commonly utilized as anesthesia with neuroprotective effect in clinical surgical settings [13–15], which cannot only reduce the incidence of POCD but also can delay onset and shorten its duration in the elderly patients [16–27]. However, there are inconsistent findings among those studies. Thus, this systematic review will assess the efficacy and safety of propofol on POCD after GA.

### Aim

The aim of this systematic review is to explore the evidence for the efficacy and safety of propofol for patients with POCD after GA.

### Objective

The objective of this study is to systematically identify studies that synthesize all available evidence on the efficacy and safety of propofol compared to other anesthesia in patients with POCD after GA and to determine the estimated clinical benefits and harms.

## Methods

### Study protocol registration

This protocol has been registered on PROSPERO (CRD42020164096), and has been reported following the Preferred Reporting Items for Systematic Reviews and Meta-Analysis Protocol (PRISMA-P) statement (Additional file 1) [28].

### Eligibility criteria

The inclusion criteria of the review are (a) trial design as randomized controlled trials (RCTs) only; (b) trials performed in adults (aged 18 years old or over) with POCD (diagnostic criteria including World Health Organization or national guidelines) after GA; (c) have defined any types of propofol as the intervention group, and any other anesthesia management as the control group; (d) trials published in any language, including English and Chinese. No time limits will be applied to the searches.

The exclusion criteria are (a) animal study, review, comment, case report, case series, non-clinical study, uncontrolled trial, and non-RCTs; (b) adolescents (aged less than 18 years old); (c) cognitive dysfunction before surgery or caused by any other diseases, such as Alzheimer’s disease; (d) patients requiring intensive care or with severe diseases; and (e) other anesthesia that may affect the efficacy of propofol.

### Information sources and search strategy

The following electronic databases will be searched from inception: Cochrane Library, MEDLINE, EMBASE, PsycINFO, Web of Science, Scopus, Allied and Complementary Medicine Database, Chinese Biomedical Literature Database, and China National Knowledge Infrastructure for the identification of studies. Besides, we will examine other literature sources, including conference abstracts, dissertations, and reference lists of relevant reviews, that may help to overcome the publication bias due to the selective availability of data. Moreover, a comprehensive Cochrane Library search strategy is developed in consultation with an experienced medical librarian and expert in literature searching (Table 1). We will adapt similar search strategy to other electronic databases.

**Table 1:** Search strategy applied in Cochrane Library database

Number	Search terms
1	MeSH descriptor: (postoperative cognitive complications) explode all trees
2	((postoperative*) or (cognitive*) or (dysfunction*) or (complications*) or (disorder*) or (surgery*) or (operation*)):ti, ab, kw
3	Or 1-2
4	MeSH descriptor: (anesthesia, general) explode all trees
5	MeSH descriptor: (propofol) explode all trees
6	((anesthesia*) or (anesthestics*) or (propofol*) or (Anesthesia S/I-60*) or (Anesthesia S/I-40*) or (Anesthesia S/I-40A*) or (Anesthesia S/I-40H*) or (Anesthesia S/I-40S*) or (Diprivan*)):ti, ab, kw
7	Or 4-6
8	MeSH descriptor: (randomized controlled trial) explode all trees
9	((random*) or (randomly*) or (blind*) or (controlled trial*) or (clinical trial*) or (control*) or (study*) or (trial*)):ti, ab, kw
10	Or 8-9
11	3 and 7 and 10

*Represents multiple characters

### Study selection

Two authors will independently perform study selection based on pre-designed eligibility criteria. All searched records will be imported into citation management system (Endnote X9), and we will filter and remove all duplicates. First, all studies will be identified by screening titles/abstracts, and irrelevant records will be eliminated. Then, the full text of potential studies will be obtained and checked against all pre-designed inclusion criteria. If divergences occur, a third author will help determine and solve them to reach a final decision about whether the trial meets the eligibility criteria through discussion or consensus meeting.

### Outcome measurements

Primary outcome includes changes of cognitive impairments from baseline (as measured by any validated scales, such as Modified Mini-Mental State Examination scale [29] and Cognitive Failure Questionnaire [30]).

Secondary outcomes consist of impairments in short-term memory (as measured by any validated scores, including Short-term Memory Summary score), concentration (as checked by any validated tools), language comprehension (as appraised by any validated scales), social integration (as examined by any validated measurements); quality of life (as assessed by validated tools); and adverse events.

### Data extraction and management

Two authors will independently extract data using a previously designed standard data extraction form. Any differences will be solved by a third author through discussion and a final decision will be reached. We will extract data by the form of study information (e.g., first author, title, country, year of publication, and sample size), patient characteristics (e.g., age, sex, and eligibility criteria), study setting, study quality (e.g., random sequence generation, allocation details, and blind), details of interventions and controls, outcome indicators, and any other relevant information. Continuous data will be presented as means, standard deviations, standard errors, and 95% confidence intervals (CIs), while dichotomous data will be exerted as frequencies and percentages (%) and 95% CIs.

### Risk of bias assessment

Two authors will independently evaluate and cross check the risk of bias of RCTs using Cochrane risk of bias tool through selection, performance, detection, attrition, reporting, and other risk of bias [31]. Each domain will be rated as low, unclear, or high risk of bias. Any discrepancy between two authors will be resolved through discussion with another experienced author.

### Strength of evidence

We will appraise strength of evidence for each outcome using Grading of Recommendations Assessment, Development and Evaluation tool (GRADE) [32, 33]. It covers risk of bias, inconsistency, imprecision, indirectness, publication bias, and other factors and related with incorporated findings on each outcome measurement properties. Two authors will independently assess the strength of evidence for each outcome. Any disagreement will be solved by a third author through consultation or discussion. The summary of all outcomes will be summarized in a table following the principle of GRADE.

### Data synthesis and statistical analysis

RevMan 5.3 software will be used to perform data synthesis and statistical analysis. Continuous data will be pooled using mean difference (MD) or standardized MD (SMD) with its respective 95% CIs. Dichotomous data will be pooled using risk ratios with its respective 95% CIs. We will apply *I*^*2*^ statistic test to check heterogeneity across included trials [34]. A value of *I*^*2*^ ≤50% will be considered to mean low heterogeneity, while the value of *I*^*2*^ >50% will be considered to suggest high heterogeneity. A random-effects model will be employed to pool the data [34].

If sufficient RCTs are available and variability across eligible trials is low, a meta-analysis will be performed according to the comparisons of different study information, patient characteristics, details of propofol and controls, and outcome indicator measurements. If obvious heterogeneity is identified, a subgroup analysis and a meta-regression analysis will be carried out to investigate the potential sources of significant heterogeneity. If meta-analysis is deemed not appropriate, descriptive statistics and narrative synthesis of data will be carried out. Furthermore, when the number of eligible trials for this review is over 10, a funnel plot will be plotted for testing reporting bias, and asymmetry of the funnel plot will be examined using Egger’s regression test [35, 36]. Sensitivity analysis will be utilized to test the robustness of study findings by eliminating low quality study.

### Amendments

Any amendments to this protocol will be recorded with reference to saved searches and analysis.

## Discussion

Although similar systematic reviews investigated the effects of propofol on POCD [18, 37], they all focused on different aspects, such as combination of propofol and inhalation anesthesia on POCD in elderly, and propofol vs. sevoflurane on POCD in elderly with LC [18, 37]. This proposed systematic review aims to appraise the efficacy and safety of propofol on POCD after GA. Any amendments with regard to the present study when performing the analysis will be highlighted and reported in the final manuscript. The present study will summarize the most recent evidence of propofol on POCD after GA. The findings of this study may provide helpful evidence for both clinical practice and health-related decision makers.

This proposed study may still suffer from several limitations at review level. First, the methodological quality of eligible studies may be poor, which may affect the robustness of the study findings. Second, insufficient number of eligible trials and its small sample size may affect the results of this study. Third, significant heterogeneity across included studies may lead to challenges in the interpretation of the results.

## Supplementary Information

**Additional file 1:.** PRISMA-P (Preferred Reporting Items for Systematic review and Meta-Analysis Protocols) 2015 checklist: recommended items to address in a systematic review protocol*

## Data Availability

Data sharing is not applicable to this article as no datasets were generated or analyzed during the current protocol.

## Abbreviations

POCD: Postoperative cognitive dysfunction
GA: General anesthesia
PRISMA-P: Preferred Reporting Items for Systematic Reviews and Meta-Analysis Protocol
RCTs: Randomized controlled trials
CIs: Confidence intervals
MD: Mean difference
SMD: Standardized MD
GRADE: Grading of Recommendations Assessment, Development and Evaluation tool
